# T2-weighted imaging and dynamic contrast‑enhanced imaging in predicting the prognosis in patients with acute-on-chronic liver failure

**DOI:** 10.1186/s12876-023-02920-2

**Published:** 2023-08-17

**Authors:** Yan Ni Du, Chun Shuang Guan, Zhi Bin Lv, Ming Xue, Yu Xue Xing, Ru Ming Xie

**Affiliations:** Department of Radiology, Beijing Ditan Hospital, Capital Medical University, No. 8 Jingshun East Street, Chaoyang District, Beijing, China

**Keywords:** Acute-on-chronic liver failure, Prognostic factors, Magnetic resonance imaging

## Abstract

**Background:**

Acute-on-chronic liver failure (ACLF) is a syndrome with high 28- and 90-day mortality rates. Magnetic resonance imaging (MRI) has been widely used to diagnose and evaluate liver disease. Our purpose is to determine the value of the imaging features derived from Gd-DTPA-enhanced MRI for predicting the poor outcome of patients with ACLF and develop a clinically practical radiological score.

**Methods:**

This retrospective study comprised 175 ACLF patients who underwent Gd-DTPA-enhanced abdominal MRI from January 2017 to December 2021. The primary end-point was 90-day mortality. Imaging parameters, such as diffuse hyperintense of the liver on T2WI, patchy enhancement of the liver at the arterial phase, uneven enhancement of the liver at the portal vein phase, gallbladder wall edema, periportal edema, ascites, esophageal and gastric varix, umbilical vein patefac, portal vein thrombosis, and splenomegaly were screened. Cox proportional hazard regression models were used to evaluate prognostic factors and develop a prediction model. The accuracy of the model was evaluated by receiver operating characteristic (ROC) curves.

**Results:**

During the follow-up period, 31 of the 175 ACLF patients died within 90 days. In the multivariate analysis, three imaging parameters were independently associated with survival: diffuse hyperintense on T2WI (p = 0.007; HR = 3.53 [1.40–8.89]), patchy enhancement at the arterial phase (p = 0.037; HR = 2.45 [1.06–5.69]), moderate ascites (vs. mild) (p = 0.006; HR = 4.12 [1.49–11.36]), and severe ascites (vs. mild) (p = 0.005; HR = 4.29 [1.57–11.71]). A practical radiological score was proposed, based on the presence of diffuse hyperintense (7 points), patchy enhancement (5 points), and ascites (6, 8, and 8 points for mild, moderate, and severe, respectively). Further analysis showed that a cut-off at 14 points was optimum to distinguish high-risk (score > 14) from the low-risk group (score ≤ 14) for 90-day survival and demonstrated a mean area under the ROC curve of 0.774 in ACLF patients.

**Conclusions:**

Gd-DTPA-enhanced MR imaging features can predict poor outcomes in patients with ACLF, based on which we proposed a clinically practical radiological score allowing stratification of the 90-day survival.

## Introduction

Acute-on-chronic liver failure (ACLF) is a syndrome that develops whereby patients with chronic liver disease (CLD) or cirrhosis rapidly develop liver function decompensation [1, 2]. The most dominant etiology of ACLF is hepatitis B virus (HBV) infection in the Asian region and alcoholism in European and American countries. HBV infection is very common in China [3, 4]. Despite precipitating events and supportive treatments, ACLF has high 28- and 90-day mortality rates [5–7]. Liver transplantation represents the only definitive and effective treatment, but its clinical application is limited due to the high mortality associated with post-transplantation, expensive medical costs and the lack of donors [8]. Hence, early diagnosis, accurate outcome prediction and timely preliminary treatments are crucial for assessing the risk-to-benefit ratio of emergency liver transplantation.

Reportedly, there are several conventional models available to estimate liver function and prognosis of patients with ACLF, widely used are the Model for End-Stage Liver Disease (MELD) score, such as the Model for End-stage Liver Disease-sodium (MELD-Na), and Child–Turcotte–Pugh (CTP) score [9–11]. Novel predictive assessment models, i.e., CLIF-C OFs, CLIF-SOFAs, and CLIF-C ACLFs, proposed by EASL-CLIF have been validated to forecast mortality in patients with ACLF [12]. However, these models mostly rely on biological metrics, require complex computations with subjective evaluation, and may be difficult to easily apply in clinical practice.

Abdominal imaging examinations have been widely used to diagnose and evaluate liver disease, especially magnetic resonance imaging (MRI) with the advantages of noninvasiveness, nonionizing radiation, and multiple parameters [13–15]. Although the underlying pathophysiology of ACLF is poorly understood, liver inflammation and subsequent systemic inflammation are believed to play a major role in the development and progression of the disease [16]. Previous studies have shown that hepatic MR imaging features are correlated with the pathophysiology of hepatitis and liver failure, and there are also related studies on imaging features for patients with ACLF [17–19]. However, to the best of our knowledge, there is no multiparametric MRI study for predicting the outcomes of patients with ACLF.

To fill this gap in the literature, the present study determined the imaging features of Gd-DTPA-enhanced MRI linked to 90-day survival and developed a simple and practical radiological score to estimate the outcomes of patients with ACLF.

## Patients and methods

### Patient cohort

**Fig. 1:**
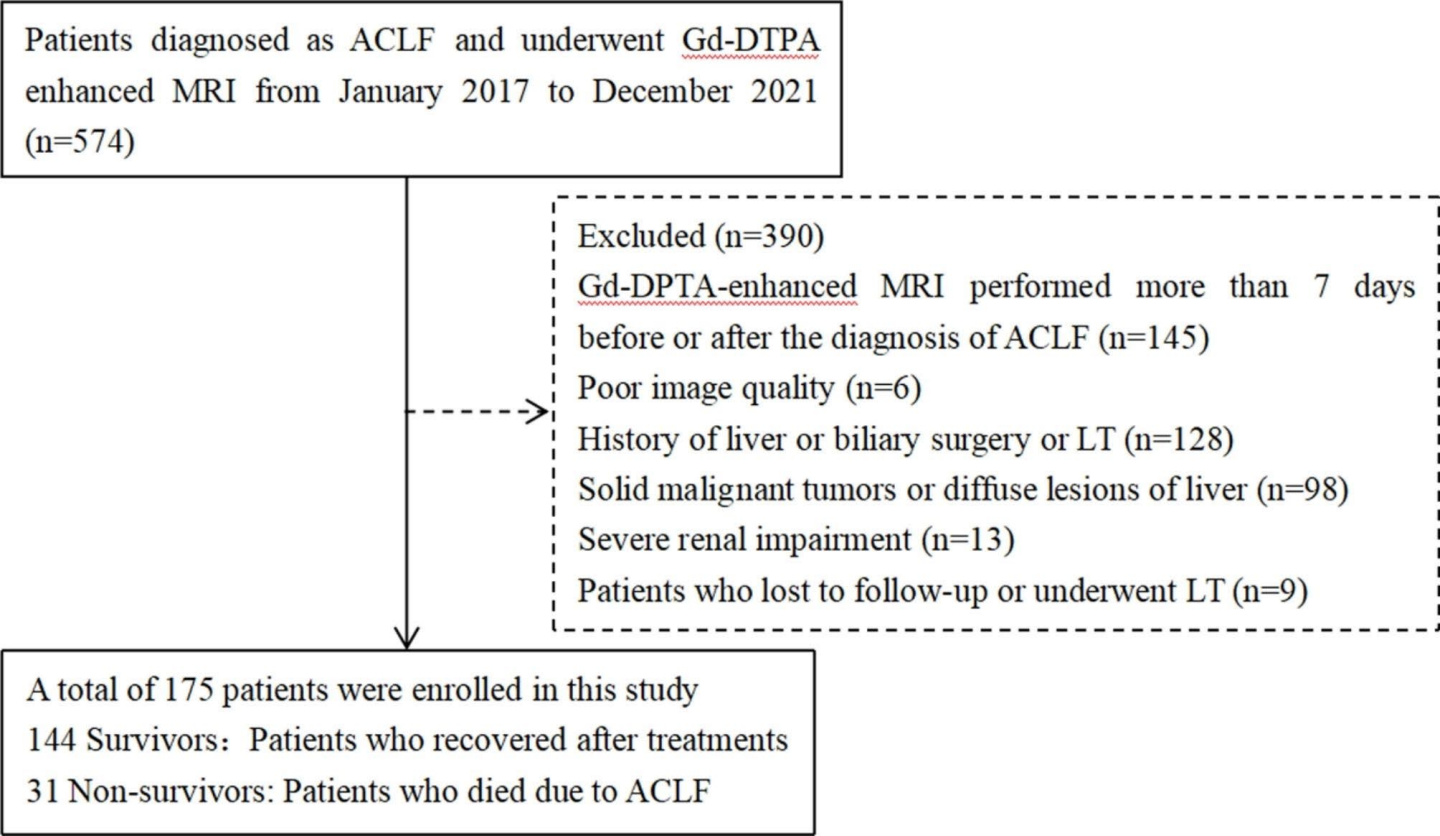
Flowchart of patient selection

In this single-center retrospective study, all of the patients who were diagnosed with ACLF and underwent abdominal enhanced MRI using Gd-DTPA (gadopentetate dimeglumine) from January 2017 to December 2021 were enrolled. The retrospective study was approved by the Bioethics Committee of Beijing Ditan Hospital, Capital Medical University (No. DTEC-KY2022-0080-01). Signed informed consent was obtained from all the patients who participated in this study. The diagnosis of ACLF was based on the Asian Pacific Association (APASL) ACLF Research Consortium (AARC) criteria, which included jaundice (total bilirubin levels ≥ 5 mg/dL), coagulation failure (INR ≥ 1.5 or prothrombin activity < 40%), complicated by evident ascites and/or encephalopathy within 4 weeks occurring in patients with chronic liver disease or cirrhosis [1]. Exclusion criteria were (i) Gd-DPTA-enhanced abdominal MRI performed more than 7 days before or after the diagnosis of ACLF and poor imaging quality, (ii) history of liver, hepatic vascular surgery or liver transplant (LT), (iii) solid malignant tumors of the liver, (iv) chronic severe renal impairment, and (v) patients who lost to follow-up or underwent LT. Finally, 175 patients were eligible for this study (Fig. 1).

All patients received treatments for precipitating events, such as bacterial or fungal infection, acute variceal hemorrhage, alcoholic hepatitis, and hepatitis B virus reactivation, and supportive therapy (such as extracorporeal liver support, treatment of encephalopathy, and cardiovascular support). The study outcome was the 90-day survival status of patients. Prognostic information for the date of death was acquired by checking electronic medical records or contacting the patient’s family.

### Clinical Data

Clinical and laboratory data were obtained from our institutional database, which included patient age, sex, serum albumin, serum creatinine, total bilirubin, international normalized ratio (INR), alanine aminotransferase (ALT), aspartate aminotransferase (AST), white blood cell (WBC), monocyte and C-reactive protein (CRP) obtained within 3 days, and hepatic encephalopathy (HE) (presence or absence) within 4 weeks before or after the diagnosis of ACLF. CTP scores were collected according to the above factors and were grouped into CTP class A (5–6 points), B (7–9 points) or C (10–15 points) [20]. The MELD score was calculated using the following formula: MELD score = 10 × (0.957 ×ln(serum creatinine) + 0.378 × ln (total bilirubin) + 1.12 × ln (INR)) + 0.643 [21].

## MRI protocols

MRI examinations were performed using a 3.0 T system (GE Healthcare Discovery 750 W) combined with a 16-element phased-array abdominal coil and a fixed spine coil. A standard dose of gadopentetate dimeglumine (0.1 mmol/kg; Magnevist, Bayer) was injected intravenously at a rate of 1.0 mL/sec and flushed with a bolus of saline (NaCl, 0.9%) at the same rate. The dynamic contrast-enhanced (DCE) sequence consisted of three-dimensional T1-weighted liver acquisition with volume acceleration (LAVA) sequences. Axial images were acquired before and in the arterial phase (AP) (25 s), portal venous phase (PVP) (70 s) and delayed phase (DP) (3 min) after contrast injection. The scanning parameters were as follows: TR/TE = 5.0/1.7 ms, flip angle = 12°, slice thickness = 4.4 mm, field of view (FOV) = 400.0 × 320.0 mm^2^, matrix = 256 × 200, bandwidth = 62.5, and acquisition time = 10 s. The other MRI examination protocol was fat-saturated respiratory triggered fast recovery fast spin echo (FRFSE) T2-weighted images (T2WI). The scanning parameters were as follows: TR/TE = 11,250/87 ms, flip angle = 90°, slice thickness = 7 mm, FOV = 380.0 × 380.0 mm^2^, matrix = 288 × 288, bandwidth = 83.3, and acquisition time = 135 s.

### Imaging analysis

**Fig. 2:**
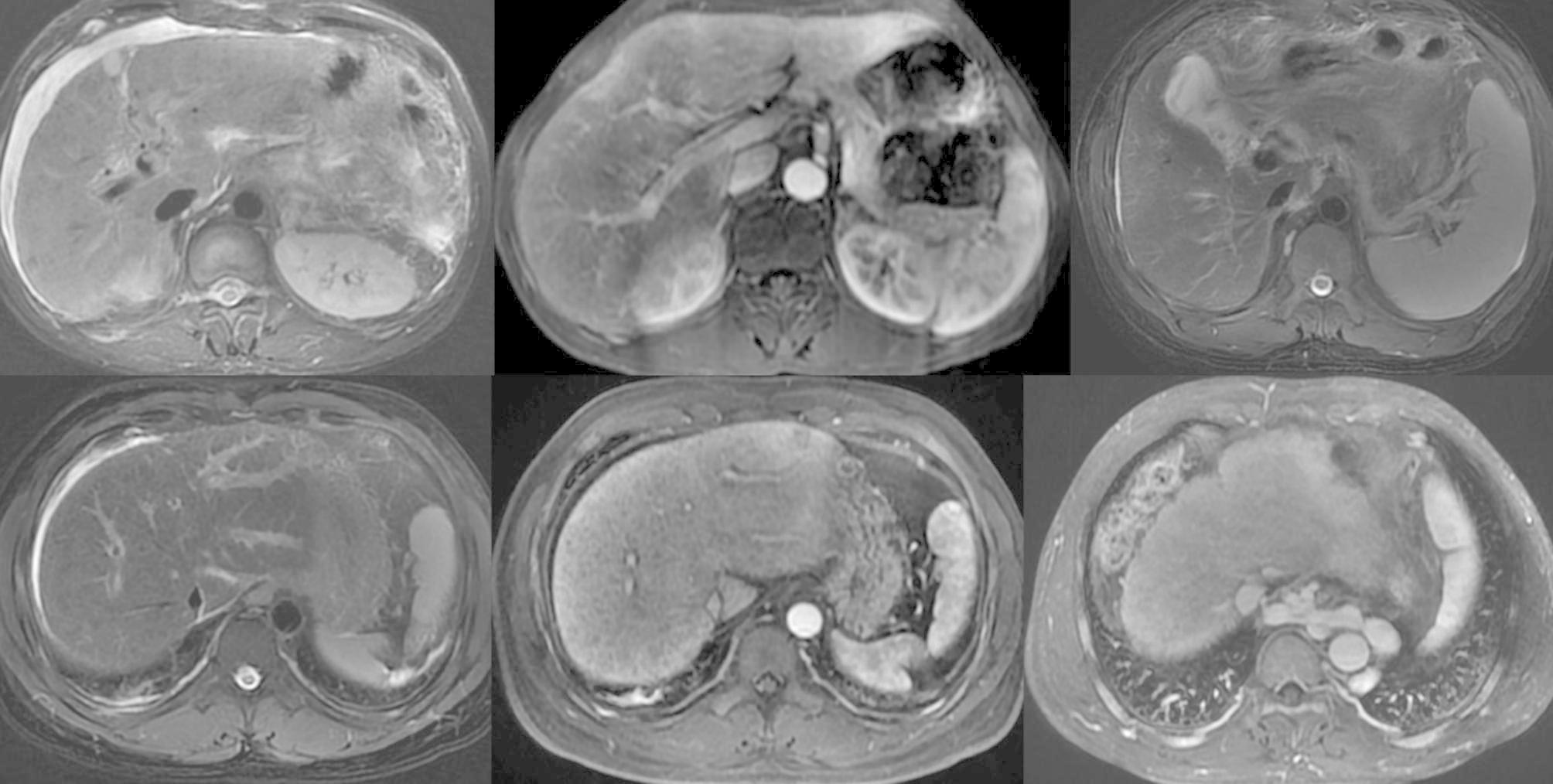
MR imaging features of ACLF. (**a**) Diffuse hyperintense of the liver on T2WI and ascites in patients with decompensated cirrhosis; (**b**) patchy enhancement of the liver mainly located in the left lobe at the arterial phase in patients with chronic hepatitis B; (**c**) gallbladder wall thickening on T2WI in patients with compensated cirrhosis; (**d**) and (**e**) periportal edema, (**d**) hyperintense tramlines or rings around the flow void in the segmental portal vein in patients with chronic hepatitis B on T2WI and (**e**) low intensity at corresponding locations at the PVP; and (**f**) gastric varix in patients with decompensated cirrhosis at the PVP.

Two radiologists with 15 and 5 years of experience in abdominal imaging reviewed and discussed the MR images in consensus. The MR imaging features of ACLF were evaluated based on previous imaging studies and clinical experience [17–19, 22], which included (a) diffuse hyperintense of the liver on T2WI, (b) patchy enhancement of the liver at the arterial phase, (c) uneven enhancement of the liver at the portal vein phase, (d) gallbladder wall edema, (e) periportal edema, (f) ascites, (g) esophageal varix, (h) gastric varix, (i) umbilical vein patefac, (j) portal vein thrombosis, and (k) splenomegaly (Fig. 2). Gallbladder wall edema was defined as hyperintensity on T2WI between the inner and outer walls. Periportal edema was defined as hyperintense tramlines or rings surrounding the portal vein and branches on T2WI or low intensity tramlines or rings around the portal vein and branches at PVP. Ascites were classified into mild, moderate, and severe according to the amount [23].

### Statistical methods

All data were analysed using SPSS 21.0 statistical software, MedCalc Statistical Software version 17.1 and R statistical software version 3.3.3. The intraclass correlation coefficients (ICCs) with their two-tailed P values were calculated for each variable predictor between two readers to determine the interobserver reliability. Continuous variables were expressed as mean ± standard deviation (SD) or median (interquartile range), and categorical variables were presented as frequencies (proportions). Continuous variables were assessed using Student’s t test or the Mann‒Whitney U test where applicable and categorical variables were assessed using the χ2 or Fisher exact test.

The 90-day patient mortality with ACLF was the primary endpoint. Cox proportional hazard regression models were used to identify the associations of imaging-based metrics and calculate the corresponding hazard ratio (HR) and 95% confidence interval (CI). Factors with a p value < 0.1 in univariate analysis were included in the multivariate regression model. For multiple comparisons in multivariate analysis, Bonferroni correction was performed.

A radiological score was then built with MR imaging parameters significantly associated with poor outcome in the multivariate analysis. The predictive accuracy of the radiological score was assessed by receiver operating characteristic (ROC) curve and area under the curve (AUC). The Youden index was used to identify the optimal cut-off point. The cumulative survival of ACLF patients was estimated using the Kaplan–Meier method. Differences were considered significant at p < 0.05.

## Results

### Patient characteristics

The data of the 175 patients with ACLF were reviewed, comprising 28 females and 147 males. The median age was 48.6 ± 12.3 (range 24–87) years. According to the follow-up results, 144 patients were grouped into the survival group and 31 patients who died within 90 days were grouped into the nonsurvival group. The demographic characteristics and laboratory data of the survival and nonsurvival groups are summarized in Table 1.

**Table 1:** Characteristics of the study population

Variables	Total (n = 175)	Non-survivors (n = 31)	Survivors (n = 144)	*p*-value
Gender (male/female)	146/29	27/4	119/25	0.545
Age (y) Mean ± SD	48.6 ± 12.3(24–87)	53.8 ± 13.6(31–79)	47.5 ± 11.8(24–87)	0.009^*^
Etiology of liver disease n (%)				0.087
Hepatitis B virus	124(64.9)	16(45.7)	108(69.2)	
Hepatitis C virus	5(2.6)	2(5.7)	3(1.9)	
Hepatitis E virus	4(2.1)	2(5.7)	2(1.3)	
Alcoholic liver disease	35(18.3)	10(28.6)	25(16)	
Primary biliary cirrhosis	4(2.1)	1(2.9)	3(1.9)	
Autoimmune hepatitis	5(2.6)	1(2.9)	4(2.6)	
Nonalcoholic steatohepatitis	5(2.6)	1(2.9)	4(2.6)	
Drug-induced hepatitis	9(4.7)	2(5.7)	7(4.5)	
Liver disease stage				0.469
CLD	44(25.1)	5(16.1)	39(27.1)	
compensated cirrhosis	24(13.7)	5(16.1)	19(13.2)	
decompensated cirrhosis	107(61.1)	21(67.7)	86(59.7)	
Biocharacteristics				
Albumin(g/L)	31.3 ± 6.3(17.1–69.4)	30.1 ± 8.8(17.1–69.4)	31.5 ± 5.6(19.7–66.5)	0.315
Creatinine(µmol/L)	71.2(59.1–84.3)	78.6(58.4-127.4)	70.0(59.1–81.8)	0.049^*^
Total bilirubin(µmol/L)	250.6 ± 125.0(87.3-614.3)	303.3 ± 149.6(96.7-614.3)	240.3 ± 120.3(87.3-553.4)	0.016^*^
INR	2.3 ± 0.6(1.5–5.4)	2.5 ± 0.9(1.6–5.4)	2.3 ± 0.6(1.5–4.5)	0.091
ALT(U/L)	230.7(65.2-752.8)	29.2(72.9-335.3)	288.9(85.8-813.4)	0.001^*^
AST(U/L)	231.5(108.2-641.2)	116.1(81.2-310.8)	251.8(125.1-670.6)	0.094
WBC (×10^9^/L)	6.3(4.3–8.5)	7.5(4.3–13.5)	6.5(4.4–8.1)	0.100
Monocyte (×10^9^/L)	0.6(0.4–0.8)	0.7(0.4-1.0)	0.6(0.4–0.8)	0.069
CRP(mg/L)	12.2(6.9–21.1)	26.1(12.3–51.0)	10.7(6.6–17.3)	<0.001^*^
HE	50(28.6)	20(64.5)	30(20.8)	<0.001^*^
CTP class				0.526
CTP B	1(3.1)	27(18.8)	28(16.0)	
CTP C	30(96.8)	117(81.3)	147(84.0)	
MELD score	23.5 ± 5.0(14.2–39.8)	27.5 ± 5.8(16.9–39.8)	22.9 ± 4.4(14.2–35.8)	<0.001^*^

Note-Data are presented as mean ± SD with ranges, median with interquartile range in parentheses, or n (%)

* *p* < 0.05 (comparing between nonsurvivors and survivors)

Abbreviations: CLD, chronic liver disease; INR, international normalized ratio; ALT, alanine aminotransferase; AST, aspartate aminotransferase; WBC, white blood cell; CRP, C-reactive protein; HE, hepatic encephalopathy; CTP, Child–Turcotte–Pugh; MELD, model for end-stage liver disease

### Correlation between imaging findings and 90-day survival status

Among the enrolled patients, five underwent abdominal unenhanced MRI examinations, and one had acute severe renal impairment. During the follow-up period, 31 of the 175 ACLF patients (17.8%) died within 90 days. The chi-square test was used to analyse the correlation between imaging factors and the 90-day survival status. The statistically significant factors are summarized in Table 2. The interobserver reliabilities of diffuse hyperintense on T2WI, patchy enhancement at AP, and ascites were excellent (ICC = 0.92 [95% confidence interval {CI} = 0.91, 0.94], 0.93 [95% CI = 0.92, 0.95], and 0.91 [95% CI = 0.89, 0.93] respectively). All the enrolled patients had ascites, and the incidence of mild, moderate, and severe ascites was 49.7%, 23.4%, and 26.9% respectively. Among the 11 relevant imaging features, the statistically significant factors were diffuse hyperintense of the liver on T2WI, patchy enhancement of the liver at AP, and ascites.

**Table 2:** MR imaging findings compared between the nonsurvival and survival groups: Chi-square test

Variables	Total (n = 175)	Nonsurvivors (n = 31)	Survivors (n = 144)	*P-*value
Diffuse hyperintense on T2WI	82(46.9)	24(77.4)	58(40.3)	<0.001^*^
Patchy enhancement at AP	85(48.6)	19(61.3)	66(45.8)	0.023^*^
Uneven enhancement at PVP	20(11.4)	3(9.7)	17(11.8)	0.899
Gallbladder wall edema	90(51.4)	16(51.6)	74(51.4)	0.849
Periportal edema	74(42.3)	10(32.3)	64(44.4)	0.441
Ascites (mild/moderate/severe)	87/41/47	6/11/14	81/30/33	0.001^*^
Esophageal varix	84(48.0)	13(41.9)	71(49.3)	0.860
Gastric varix	60(34.3)	11(35.5)	49(34.0)	0.535
Umbilical vein patefac	53(30.3)	8(25.8)	45(31.3)	0.832
Portal vein thrombosis	11(6.3)	2(6.5)	9(6.3)	0.836
Splenomegaly	100(57.1)	18(58.1)	82(56.9)	0.667

* *p* < 0.05 (comparing between nonsurvivors and survivors)

AP, arterial phase; PVP, portal venous phase

### Imaging parameters can predict poor outcome in ACLF patients

To determine the imaging-related predictive factors associated with the prognosis of ACLF patients, 11 imaging characteristics were included in the univariate Cox regression. The results showed that diffuse hyperintense of the liver on T2WI, patchy enhancement of the liver at AP, and ascites were significantly associated with poor prognosis (Table 3). In the multivariate analysis, these three imaging parameters were independently associated with survival as well as diffuse hyperintense of the liver on T2WI (p = 0.007; HR = 3.53 [1.40–8.89]), patchy enhancement of the liver at AP (p = 0.037; HR = 2.45 [1.06–5.69]), moderate ascites (vs. mild) (p = 0.006; HR = 4.12 [1.49–11.36]), and severe ascites (vs. mild) (p = 0.005; HR = 4.29 [1.57–11.71]) (Table 3).

**Table 3:** Cox regression analysis of MR imaging predictors for prognosis in patients with ACLF

Variables	Univariate analysis		Multivariate analysis	
	HR (95% CI)	*P* value	HR (95% CI)	*P-* value
Diffuse hyperintense on T2WI	2.15(1.03–4.49)	0.041	3.53(1.40–8.89)	0.007
Patchy enhancement at AP	2.54(1.11–5.80)	0.027	2.45(1.06–5.69)	0.037
Uneven enhancement at PVP	0.92(0.28–3.06)	0.894		
Gallbladder wall edema	1.09(0.52–2.26)	0.828		
Periportal edema	0.73(0.34–1.60)	0.436		
Moderate vs. mild	4.23(1.57–11.45)	0.004	4.12(1.49–11.36)	0.006
Severe vs. mild	4.86(1.87–12.65)	0.001	4.29(1.57–11.71)	0.005
Esophageal varix	0.94(0.44-2.00)	0.869		
Gastric varix	1.26(0.59–2.72)	0.552		
Umbilical vein patefac	0.89(0.39–2.03)	0.781		
Portal vein thrombosis	1.11(0.26–4.67)	0.890		
Splenomegaly	1.16(0.55–2.45)	0.702		

### Development of MR imaging feature-based radiological score

**Fig. 3:**
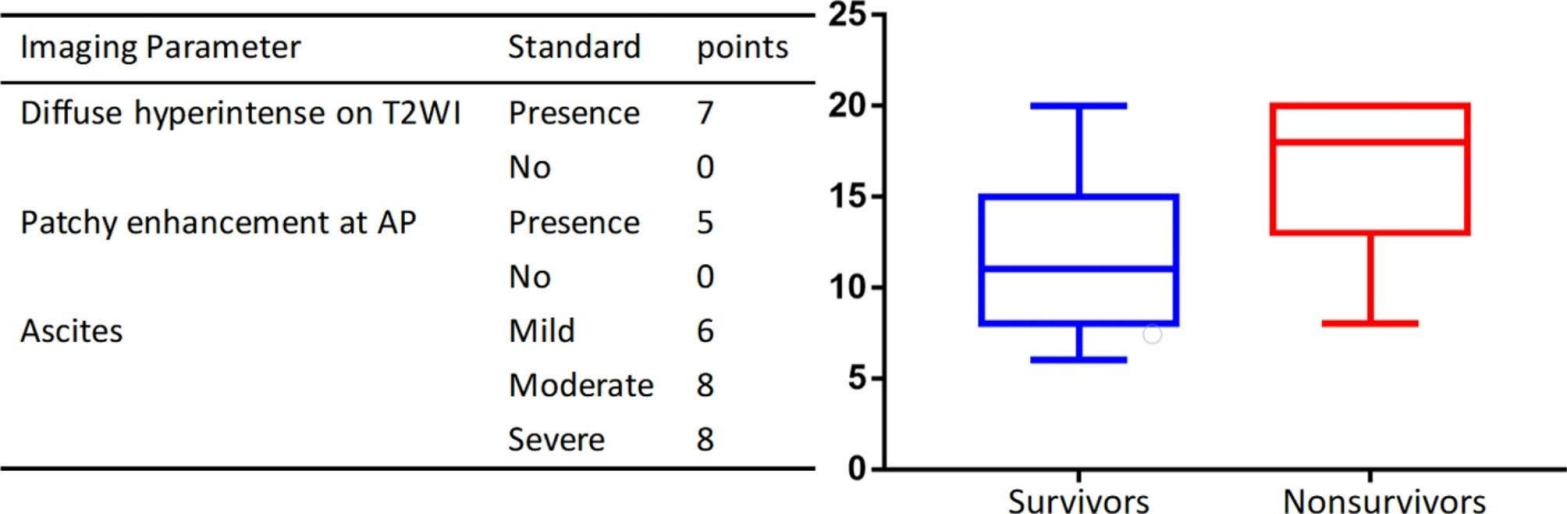
A 3-item radiological score associated with 90-day survival. (**a**) A radiological score based on the 3 imaging parameters. (**b**) Boxplots of the radiological score between the survivors and nonsurvivors

**Fig. 4:**
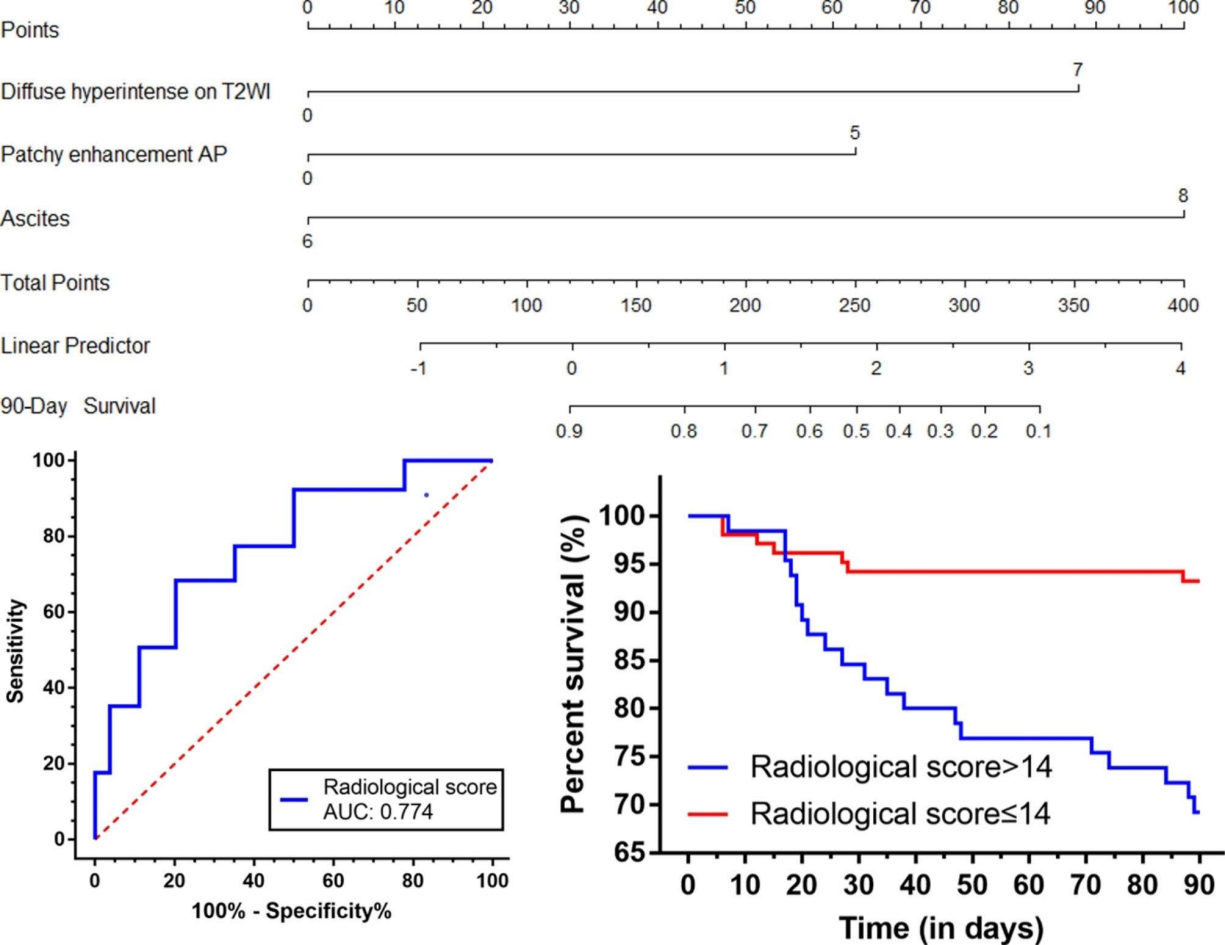
A nomogram and ROC curves and survival analysis of the radiological score model. (**a**), A nomogram was developed based on the three MR imaging features; (**b**) ROC curve of the radiological score for predicting 90-day mortality in ACLF patients in the deriving cohort; (**c**) Survival analysis using the radiological score

A radiological score including significant parameters associated with ACLF patient outcomes in multivariate analysis was developed according to their respective regression coefficients. These three imaging features were diffuse hyperintense of the liver on T2WI, patchy enhancement of the liver at AP, and ascites, given out of 20 points (Fig. 3a). The radiological score for the 90-day nonsurvivors was significantly higher than that for the survivors (16.5 ± 4.0 vs. 12.0 ± 4.6, P < 0.001) (Fig. 3b). Thus, the MR imaging feature-based radiological score model was developed to predict the risk probability of 90-day survival and presented as a nomogram (Fig. 4a).

### Performance and evaluation of the radiological score for predicting prognosis

The ROC curves of the radiological score for predicting the poor prognosis of ACLF patients are shown in Fig. 4b. The mean AUC based on data from the deriving cohort was 0.774 (95% CI, 0.681-867; P < 0.001). The sensitivity and specificity were 74.1% and 68.3%, respectively, for the cut-off value of 14. We further identified the patients based on the most appropriate cut-off value of 14 to plot the Kaplan‒Meier survival curves (Fig. 4c). The results showed that ACLF patients with radiological scores > 14 had a low 90-day cumulative survival (P < 0.001).

## Discussion

In the present study we evaluated the prognostic value of radiographic findings of multiparametric gadopentetate dimeglumine-enhanced abdominal MRI. Our study confirmed that diffuse hyperintense of the liver on T2WI, patchy enhancement of the liver at the arterial phase and ascites were independent predictors for poor prognosis in patients with ACLF. To the best of our knowledge, this is the first study using sensitive and noninvasive MR imaging features to visually evaluate the prognosis of ACLF patients, which could be used to assist decision-making for early supportive therapy and distinguish patients who need transplantation [16, 24].

Our analysis of MR imaging features showed that liver parenchyma abnormalities as diffuse hyperintense on T2WI and patchy enhancement during the arterial phase, gallbladder wall edema, periportal edema, esophageal varix, and splenomegaly were relatively common (42.3%~57.1%) in patients with ACLF. Ascites was observed in all patients, the incidence of which was significantly higher than that in patients with CLD and cirrhosis described in previous studies [17–19]. In addition, we found that age, creatinine, total bilirubin, ALT, CRP, MELD score, and HE were different between nonsurvivors and survivors of ACLF patients, which is consistent with previous findings [25, 26].

In this study, multivariate Cox regression showed that ascites (especially severe) was an independent risk factor for poor outcome in patients with the highest HR (severe vs. mild, HR = 4.29). A greater amount of ascites was associated with higher mortality of ACLF patients within 90 days. Another study by Balcar L et al. [27], which focused on the event of ascites, revealed that moderate or severe ascites (especially severe) with ACLF had extremely high short-term mortality rates. Our finding is directly in line with the above finding. Ascites indicates the severity of portal flow obstruction and portal hypertension because it could be caused by excessive vasodilator production due to portal flow obstruction [28]. Some studies have reported that hyperintense on T2WI and patchy enhancement during the arterial phase of the liver were correlated with ACLF [18, 19], but they did not look into the impact of these MR imaging appearances on the patient’s prognosis and are thus different from our study. In our study, diffuse hyperintense of the liver on T2WI and patchy enhancement of the liver during the arterial phase (HR = 3.53 and 2.45, respectively) were also independent prognostic factors of 90-day mortality in patients with ACLF. These findings were expected because the abnormal findings on MRI of liver parenchyma are based on the pathophysiological features of ACLF. Systemic inflammation and associated ‘cytokine storm’ are considered the main drivers of ACLF [29, 30]. Acute liver cell injury resulting from these inflammatory changes can lead to an increase of tissue-free water content and capillary leak. This pathophysiological process cause hyperintensity on T2WI of the liver parenchyma and was confirmed by histopathology [18, 19, 31]. DCE-MRI can provide information on hepatic parenchymal blood supply and extracellular space. The transient increasing blood supply of the hepatic artery can lead to patchy enhancement of the liver during the arterial phase of ACLF, which was correlated with liver inflammatory activity [18]. Kanematsu M et al. [32] also found that patients with patchy enhancement during the arterial phase had large amounts of inflammatory infiltration and hepatocyte necrosis in the liver, which might reflect the current or most recent liver cell damage. Moreover, a higher ACLF severity was found to be associated with higher plasma proinflammatory cytokine or chemokine levels, a higher incidence of diffuse hyperintense on T2WI and patchy enhancement during the arterial phase and poor prognosis.

Our study is the first to show that a radiological score including 3 imaging features as ascites, diffuse hyperintense on T2WI and patchy enhancement during the arterial phase of the liver could predict 90-day survival in ACLF patients. The radiological score had satisfactory prognostic ability of poor outcome with high prediction efficiency (0.774, 74.1%, 68.3% for AUC, sensitivity, and specificity, respectively). Patients with a radiological score > 14 showed a lower 90-day cumulative survival than patients with a score ≤ 14. Thus, the radiological score could be an excellent assessment tool to help clinicians intuitively and objectively evaluate patients’ prognoses from routinely performed MR imaging examinations.

This study has some deficiencies. First, this was a single-center study. Although the three imaging features and the radiological score for predicting poor outcomes in ACLF patients were developed and validated internally, multicentre retrospective or even further prospective cohort studies with a large number of cases are still needed to validate these findings. Second, since the patients included in this retrospective study were treated by different clinicians, the influence of different therapies on the prognosis of ACLF patients cannot be ruled out. Third, diffuse hyperintense and patchy enhancement of the liver are categorical variables, and ascites represents semiquantitative data, although they were intuitive and simple. However, quantitative analysis of these imaging features combined with clinical data to evaluate the poor prognosis of ACLF patients may yield improved results. Fourth, liver volume was an independent factor for short-term mortality in patients with ACLF [26], and further clinical studies are needed together with liver volume to validate and update these results.

## Conclusions

In conclusion, diffuse hyperintense of the liver on T2WI, patchy enhancement of the liver in the arterial phase, and ascites through routine abdominal enhanced MRI were identified as the three independent 90-day mortality predictors of ACLF patients. Based on the above findings, we successfully developed and internally validated a novel prediction radiological score model with favorable performance. This simple and intuitive evaluation model could be used to predict the risk of short-term mortality in patients with ACLF and support clinical decision-making for personalized treatment. In the future, a combined model which consists of MR imaging features and clinical findings may be developed to detect the high-risk population with ACLF patients robustly.

## Data Availability

All data generated or analysed during this study are included in this published article.

## Abbreviations

ACLF: acute-on-chronic liver failure
CLD: chronic liver disease
HBV: hepatitis B virus
CTP: Child-Turcotte-Pugh
MELD: Model for End-Stage Liver Disease
MRI: magnetic resonance imaging
Gd-DTPA: gadopentetate dimeglumine
INR: International normalized ratio
LT: liver transplant
ALT: alanine aminotransferase
AST: aspartate aminotransferase
WBC: white blood cell
CRP: C-reactive protein
HE: hepatic encephalopathy
DCE: dynamic contrast-enhanced
LAVA: liver acquisition with volume acceleration
AP: arterial phase
PVP: portal venous phase
DP: delayed phase
FOV: field of view
FRFSE: fast recovery fast spin echo
T2WI: T2-weighted images
SD: standard deviation
HR: hazard ratio
CI: confidence interval
ROC: receiver operating characteristic
AUC: area under the curve
